# Microscopic Characterization of the Brazilian Giant Samba Virus

**DOI:** 10.3390/v9020030

**Published:** 2017-02-14

**Authors:** Jason R. Schrad, Eric J. Young, Jônatas S. Abrahão, Juliana R. Cortines, Kristin N. Parent

**Affiliations:** 1 Department of Biochemistry and Molecular Biology, Michigan State University, East Lansing, 48824 MI, USA; schradja@msu.edu (J.R.S.); younge16@msu.edu (E.J.Y.); 2 Laboratório de Vírus, Departamento de Microbiologia, Instituto de Ciências Biológicas, Universidade Federal de Minas Gerais, Belo Horizonte, 31270-901 Minas Gerais, Brazil; jonatas.abrahao@gmail.com; 3 Unité de Recherche sur les Maladies Infectieuses et Tropicales Emergentes (URMITE) UM63 CNRS 7278 IRD 198 INSERM U1095, Aix-Marseille University, 13385 Marseille Cedex 05, France; 4 Departamento de Virologia, Instituto de Microbiologia Paulo de Góes, Universidade Federal do Rio de Janeiro, Rio de Janeiro, 21941-902 Rio de Janeiro, Brazil

**Keywords:** *Mimiviridae*, cryo-electron microscopy, cryo-electron tomography, fluorescence microscopy, scanning electron microscopy, giant viruses, nucleocytoplasmic large DNA virus (NCLDV), *Megavirales*, mimivirus

## Abstract

Prior to the discovery of the mimivirus in 2003, viruses were thought to be physically small and genetically simple. Mimivirus, with its ~750-nm particle size and its ~1.2-Mbp genome, shattered these notions and changed what it meant to be a virus. Since this discovery, the isolation and characterization of giant viruses has exploded. One of the more recently discovered giant viruses, Samba virus, is a *Mimivirus* that was isolated from the Rio Negro in the Brazilian Amazon. Initial characterization of Samba has revealed some structural information, although the preparation techniques used are prone to the generation of structural artifacts. To generate more native-like structural information for Samba, we analyzed the virus through cryo-electron microscopy, cryo-electron tomography, scanning electron microscopy, and fluorescence microscopy. These microscopy techniques demonstrated that Samba particles have a capsid diameter of ~527 nm and a fiber length of ~155 nm, making Samba the largest *Mimivirus* yet characterized. We also compared Samba to a fiberless mimivirus variant. Samba particles, unlike those of mimivirus, do not appear to be rigid, and quasi-icosahedral, although the two viruses share many common features, including a multi-layered capsid and an asymmetric nucleocapsid, which may be common amongst the *Mimiviruses*.

## 1. Introduction

Historically, following the discovery that the causative agent of tobacco mosaic disease could pass through sterile (0.22 µm) filters [1], viruses were thought to be small and simple; containing only a few genes [2]. However, the re-classification of *Acanthamoeba polyphaga mimivirus* (APMV) [3,4] fundamentally changed our understanding of viral life [5]. Originally isolated in 1993 from a water cooling tower in Bradford, UK following a pneumonia outbreak, the so-called “Bradford coccus” was initially classified as a bacteria. These supposed cocci were visible under the light microscope and appeared to stain Gram positive [3]. At the time, APMV was thought to be too large to be a virus (~700-nm particle diameter). It was not until 2003 that the inability to culture the Bradford coccus, and its lack of a 16S ribosomal RNA sequence, lead to the re-classification of this organism as the *mi*crobe-*mi*micking (Mimi) virus [3].

Since then, dozens of other “giant” viruses, defined as viruses that are readily visible through light microscopy [6], have been discovered through co-culturing with *Acanthamoeba spp.* [3,6,7]. Some of these newly discovered giant viruses fall into the viral families *Mimiviridae* [6,8] and *Marseilleviridae* [7,9], and many more remain unclassified, including Pandoravirus [10,11], Pithovirus [12], Faustovirus [13,14], and Mollivirus [15]. Of these, *Mimiviridae* has been the most well-studied [8,16,17], and APMV is the only *Mimivirus* with detailed structural information available [18,19,20,21]. A three-dimensional reconstruction of APMV (EMD-5039) [21] shows that these viruses are comprised of a multi-layered capsid, an external layer of fibers, and an internal, genome-containing nucleocapsid [20,21]. In addition, structural data has elucidated that APMV releases its genome through a unique vertex, initially termed the “stargate” [22], which is closed by a protein complex called the “starfish” seal [21]. This unique vertex opens the capsid and releases the genome-containing nucleocapsid from within the virion.

One of the newer members of *Mimiviridae*, Samba virus (SMBV) was originally isolated from the Rio Negro, a tributary of the Amazon River, in Brazil [23]. SMBV contains a ~1.2-Mbp double-stranded DNA genome, encoding for ~971 putative open reading frames [24]. All known members of *Mimiviridae* infect *Acanthamoeba spp.* [8], and SMBV, specifically, infects *Acanthamoeba castellanii*. Once the viral infection process has begun, SMBV takes over the *A. castellanii* cellular machinery and creates a viral factory within the host cytoplasm [25,26]. Similar to APMV and its Sputnik virophage [27], SMBV has an associated virophage, Rio Negro virus [23].

As the prototypical member of *Mimiviridae*, and the first giant virus to be characterized, APMV has become the standard to which all subsequent members of this viral family are compared. As SMBV and APMV are both members of *Mimiviridae*, it is likely that the two viruses share some structural features. Some of these common features, including a multi-layered capsid, external fibers, etc., were observed during the initial isolation and characterization of SMBV particles [23]. This original study utilized thin-section transmission electron microscopy (TEM) to generate a first glimpse of the structural features of the SMBV virion, estimating the total particle size (capsid + fibers) at ~575 nm. While this initial characterization provided invaluable structural and biological information about SMBV, the sample preparation techniques used during sectioning of biological samples are prone to the generation of structural artifacts [28,29].

To obtain a more native-like view of the structural features present in the SMBV virion, we analyzed SMBV particles through the use of cryo-electron microscopy (cryo-EM), cryo-electron tomography (cryo-ET), scanning electron microscopy (SEM), and fluorescence light microscopy. The vitrification process utilized during sample preparation for cryo-EM and cryo-ET [29] preserve the viral particles in a near-native state, limiting the generation of structural artifacts. While not as artifact-free as vitrification, the critical point drying technique used during the preparation of SEM samples avoids dehydration and physical shearing of particles that accompanies thin section sample preparation, providing more native-like structural information. Fluorescence light microscopy relies on the addition of fluorescent dyes, which may result in the generation of some structural artifacts, but this process retains specimens in a fully hydrated state.

To compare SMBV and APMV, we analyzed a fiberless variant of APMV [30] through the use of cryo-EM, SEM, and fluorescence microscopy. Two differences were readily apparent between SMBV and APMV; SMBV appeared to be less structurally rigid than the quasi-icosahedral particles of APMV, and the SMBV virion was larger than that of APMV. SMBV particles displayed a high level of structural heterogeneity and appeared to deviate from quasi-icosahedral symmetry in the cryo-electron and scanning electron micrographs. SMBV had a larger capsid (by ~27 nm), and longer fibers (by ~30 nm) than those of APMV (500-nm capsid diameter, 125-nm fiber length) [20], making SMBV the largest known *Mimivirus*. Aside from these readily visible differences, SMBV and APMV shared many common features, including the presence of multiple layers of the viral capsid, an external layer of fibers, etc. Given the relatedness of SMBV and APMV, we propose that the structural characteristics demonstrated here may be common amongst *Mimiviridae*.

## 2. Materials and Methods

### 2.1. Virus Preparation

The giant viruses were both propagated following the same protocol. *A. castellanii* cells were cultured in 712 PYG w/Additives (ATCC), at pH 6.5, in the presence of the antibiotics gentamicin and penicillin/streptomycin, with final working concentrations of 15 μg/mL and 100 U/mL, respectively, to reach a 90% confluence. Cells were then counted using a Newbauer chamber and a solution of APMV or SMBV (diluted in PBS (phosphate buffered saline), just enough to cover the cell monolayer) was added to a multiplicity of infection (M.O.I.) of 10 for 1 h at room temperature. After the incubation was finished, PYG media was added in the presence of the antibiotics (above) and culture flasks were incubated at 28 °C for 48 h, when most of the amoebal cells were lysed as a result of the infection. The suspension containing cell debris and cell particles were centrifuged at 900× *g*; the resulting supernatant was carefully filtered using a 2-μm filter and then was immediately applied over a 22% sucrose cushion (*w*/*w*) at 15,000× *g* for 30 min. Visible white viral particle pellets were resuspended in PBS and stored at −80 °C. Viruses were titered using the Reed–Muench protocol [31]. On average, virus isolation yielded 10^10^ TCID_50_/mL (TCID = tissue culture infective dose).

### 2.2. Preparation of Cryo Specimens

Small (5 µL) aliquots of purified virus particles (either APMV or SMBV) were vitrified using established procedures [29]. Samples were applied to holey Quantifoil grids (R3.5/1), which had been plasma cleaned for 20 s in a Fischione model 1020 plasma cleaner. Grids were blotted for 7–10 s using Whatman filter paper to remove excess sample, plunged into liquid ethane for vitrification, and then transferred to a pre-cooled Gatan 914 specimen holder, which maintained the specimen at liquid nitrogen temperature.

### 2.3. Low-Dose Imaging Conditions

Virus particles were imaged in a JEOL JEM-2200FS TEM operating at 200 keV, using low-dose conditions controlled by SerialEM (v3.5.0_beta) [32] with the use of an in-column Omega Energy Filter, operating at a slit width of 35 eV. Micrographs were recorded using a Direct Electron DE-20 camera (Direct Electron, LP, San Diego, CA, USA), cooled to −40 °C. Movie correction was performed on whole frames using the Direct Electron software package, v2.7.1 [33]. Micrographs used for single particle analysis were recorded on the DE-20 using a capture rate of 25 frames per second for a total exposure ranging from 75 to 300 frames (~35 e^-^/Å^2^ total dose recorded at the DE-20 sensor). Cryo-EM images were acquired between 4000 and 20,000× nominal magnifications (14.7–2.61 Å/pixel, respectively). The objective lens defocus settings for single particle images ranged from 15 to 25 µm underfocus.

### 2.4. Cryo-Electron Tomography

After plasma cleaning, but prior to the addition of SMBV particles, 5 µL of a solution of 10 nm nanogold fiducial markers were air-dried onto holey carbon grids. Tilt series projections were acquired using SerialEM (v3.5.0_beta) [32] at a capture rate of 15 frames per second for 45 frames per tilt angle, along a tilt range of ±55° with tilt increments of 1–2° and 0.7 electrons per square angstrom per tilt image. Tilt series were acquired at 4000 or 8000× nominal magnification (14.7 or 6.87 Å/pixel). Tilt series alignment was performed using IMOD (v4.7.15) [34] and standard tomographic reconstruction practices, using both the SIRT (simultaneous iterative reconstruction) and WBP (weighted back projection) reconstruction strategies. The contrast in the tomograms generated using SIRT was far better than the contrast in the tomograms generated using the WBP reconstruction strategy, therefore we have presented the SIRT data here. Contrast was increased in the tomograms through median (x3) and Gaussian (1.5 pixels) filtering. Key features of the tomograms were traced using the drawing tools functionality in IMOD (3dmod).

### 2.5. Fluorescence Microscopy

APMV and SMBV particles were stained with 1 μg/mL 4’,6-diamino-phenylindole (DAPI, DNA) and 0.1 μg/mL fluorescein isothiocyanate (FITC, protein) overnight. Virus particles were then imaged using a Zeiss Axio Observer A1 microscope (100×, 1.45 NA) outfitted with an Axiocam ICc5 camera. DAPI fluorescence was imaged with Zeiss filter set 49 and FITC fluorescence was imaged with Zeiss filter set 38 HE. Micrographs were then processed using Zeiss Zen software.

### 2.6. Scanning Electron Microscopy

SMBV particles were imaged using the in-lens detector of a JEOL JSM-7500F (SMBV) or a FEG Quanta 200 FEI (APMV) scanning electron microscope; operating at 5 kV (JSM-7500F) or 15kV (Quanta 200). Prior to imaging, virus particles were desiccated using an EM CPD300 critical point dryer, fixed with glutaraldehyde in PBS buffer at pH = 7.4 onto poly-l-Lysine treated SEM slides, and sputter coated with a ~2.7-nm layer of iridium using a Q150T Turbo Pumped Coater. Particles were imaged between 7000× and 50,000× nominal magnification.

### 2.7. Capsid and Nucleocapsid Measurements

**Figure 1:**
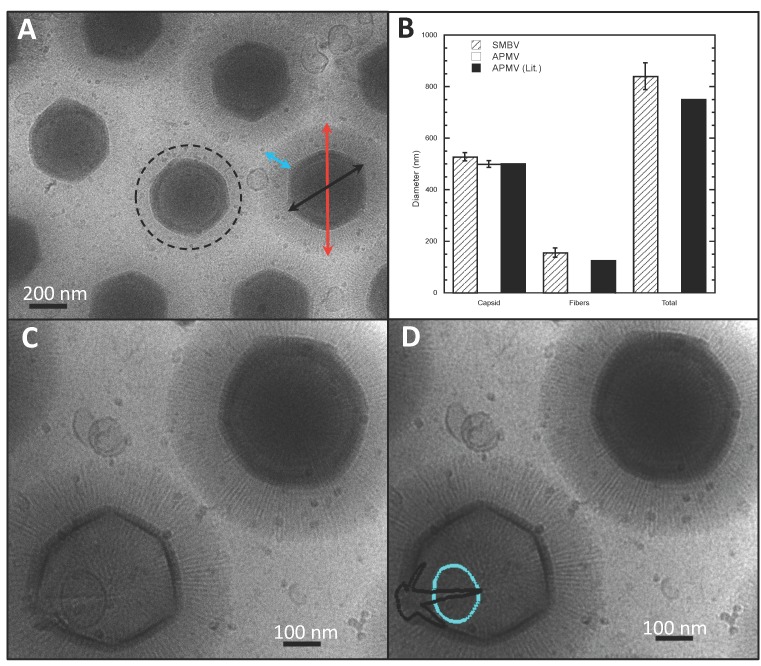
Cryo-electron microscopy data from Samba virus (SMBV) particles. (**A**) Representative micrograph depicting “fibered” and “fiberless” (circled) SMBV particles. Arrows provide an example of how the total particle diameter (red arrow), capsid diameter (black arrow), and the fiber length (cyan arrow) were measured for SMBV and *Acanthamoeba polyphaga mimivirus* (APMV) particles. (**B**) Capsid diameter, fiber length, and total particle diameter of SMBV (striped) and APMV (white) particles from this study, as well as APMV particles from Xiao et al. [20] (black). (**C**) Cryo-electron micrograph of “fibered” and “open/empty” SMBV particles. The star-shaped capsid opening (black) and the membrane sac that remains within “open/empty” particles (cyan) are highlighted in (**D**).

Capsid and total particle diameters of APMV (274 particles from 94 micrographs) and SMBV (500 particles from 226 micrographs) were measured from two-dimensional projections of cryo-electron micrographs. Capsid diameter and total particle diameter measured across three axes (putative five-fold to five-fold) for each viral particle were analyzed (Figure 1A). The length of the SMBV fibers was determined by subtracting the capsid diameter from the total particle diameter and dividing by two. All other measurements were taken using three-dimensional volumes resulting from cryo-electron tomograms. The spacing of the SMBV capsid layers was measured (11 total tomograms). Nucleocapsid dimensions could only be conclusively measured in 8 out of the 34 total tomograms, owing to contrast limitations. Nucleocapsid diameter was measured along four axes with one axis bisecting the portion of the nucleocapsid that is pulled away from the capsid, and another axis normal to the bisecting axis. The distance from the nucleocapsid to the innermost layer of the capsid was measured at the pulled away region. Capsid spacing and the distance between the nucleocapsid and the capsid were measured at ten locations throughout the remainder of the virion, in order to obtain average values throughout the SMBV capsid. All measurements were taken using the measure tool in EMAN2’s e2display.py GUI [35].

## 3. Results

### 3.1. Cryo-Electron Microscopy (Cryo-EM) Revealed the Size and Morphologies of SMBV Particles

SMBV particles, like those of all members of *Mimiviridae*, are very large, requiring a thick layer of vitreous ice (> 1 μm) to preserve the specimen in a near-native state for cryo-EM imaging. The thickness of the ice layer detracted from the contrast of SMBV cryo-EM images, especially while using a 200-keV TEM. With the use of an in-column Omega Energy Filter (JEOL 2200-FS) and a DE-20 direct detection device (Direct Electron, LP, San Diego, CA, USA), contrast in the cryo-electron micrographs was improved. SMBV particles were also imaged using a 300-keV TEM (JEOL 3200, data not shown), but these images displayed no appreciable difference in quality from the micrographs collected at 200 keV using the Omega Energy Filter. We were able to generate two-dimensional projection images of vitrified SMBV particles with sufficient contrast to accurately measure and describe several structural features of interest. The cryo-electron micrographs revealed external fibers, at least two capsid layers, and an internal genome-containing nucleocapsid within the SMBV virion (Figure 1).

Within the cryo-EM images, three distinct particle morphologies were visible, the most abundant of which were “fibered” SMBV particles (Figure 1A,B). These particles, comprising ~81% of the ~2800 particles imaged via single-particle cryo-EM, were surrounded by a layer of external fibers, which are thought to be important for host attachment. “Fiberless” particles represented the second most abundant particle morphology, at ~13.5%, (Figure 1A, indicated by a dashed circle). These do not contain external fibers. The ability of these particles to infect *A. castellanii* is currently unknown. In *Mimiviridae,* fibers are hypothesized to play a role in cell attachment and entry via phagocytosis [36], and the same may also be true for SMBV. However, a fiberless variant, “M4”, was shown to enter and propagate inside cells [30]. The least abundant particle morphology, at ~5.5% of particles, were “open/empty” SMBV particles (Figure 1B). These particles contained neither the nucleocapsid nor the double-stranded DNA genome, and were visually represented in the cryo-electron micrographs as lighter particles, due to the absence of the electron-dense material within the capsid (Figure 1C). It was hypothesized that these particles reflect a post-genome ejection stage and have opened their capsids at a unique capsid vertex (Figure 1D, highlighted in black), reminiscent of the starfish vertex seen in mimivirus [19,20,21,22]. The open/empty particles appeared to have a residual membrane component, which remained associated with the capsid after genome release (Figure 1D, highlighted in cyan). A similar residual membrane can be seen in two-dimensional projections of open APMV particles [21].

Even with low contrast, the cryo-EM images provided an accurate determination of the native size of the SMBV capsid and external fibers. The initial characterization of SMBV utilized plastic-embedded thin sections of infected amoeba and reported a capsid diameter of 352 nm, a fiber length of 112 nm, and a total particle diameter of 574 nm [23]. As mentioned previously, the sample preparation techniques used to generate thin sections of biological samples can lead to the generation of artifacts; in particular, the dehydration steps can lead to shrunken particles [28,29]. Since specimens in cryo-EM remain fully hydrated, we measured the diameter of the capsid and the total particle diameter of 500 SMBV particles to determine the size of the SMBV virion (Figure 1A,C). Averaging these measurements yielded a capsid diameter of ~527 nm (Figure 1A, black arrow) and a total particle diameter of ~834 nm (Figure 1A, red arrow), which is significantly larger than previously reported [23]. The size discrepancy between the particles visualized by cryo-EM and by thin-section TEM is most likely due to dehydration-linked particle shrinkage during the thin-section preparation steps. We were able to subtract the measured capsid diameter from the measured total particle diameter of each particle to estimate the “diameter” of the external fiber layer (assumed to be twice the fiber length). For the 500 SMBV particles measured in this study, the average fiber length (Figure 1A, cyan arrow) measured ~155 nm.

**Figure 2:**
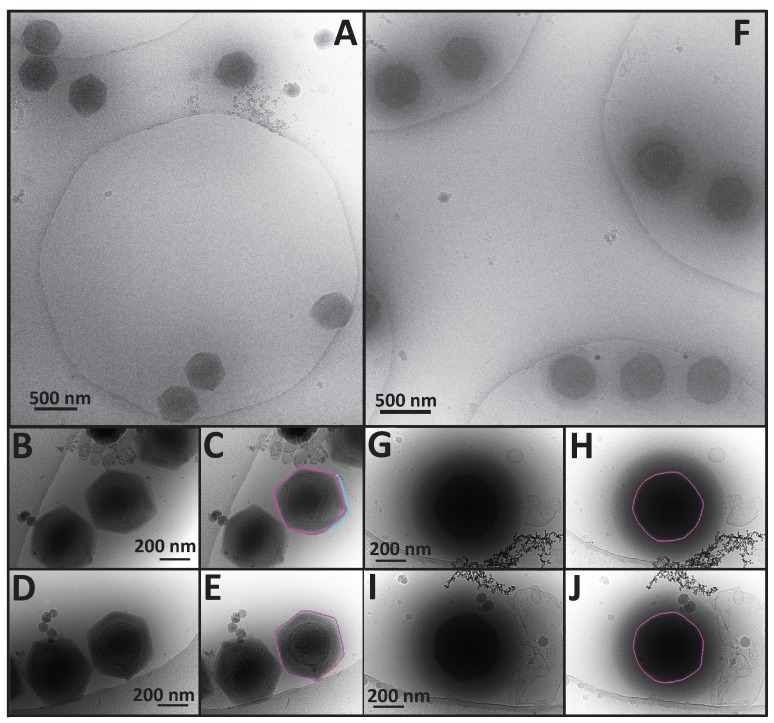
Comparison of APMV and SMBV via cryo-electron microscopy (cryo-EM) reveals that SMBV is a not rigid quasi-icosahedron like APMV, and displays a larger degree of structural variation than APMV. (**A**) Low magnification (4000×) micrograph of APMV particles. (**B**,**D**) Higher magnification (20,000×) micrographs of APMV particles with features highlighted in (**C**,**E**), respectively. (**F**) Low magnification (4000×) micrograph of SMBV particles. (**G**,**I**) Higher magnification (20,000×) micrographs of SMBV particles with features highlighted in (**H**,**J**), respectively. For panels (**C**,**E**,**H**,**J**) outer capsid layers are highlighted in magenta. The presumed starfish seal complex in panel C is highlighted in cyan.

**Figure 3:**
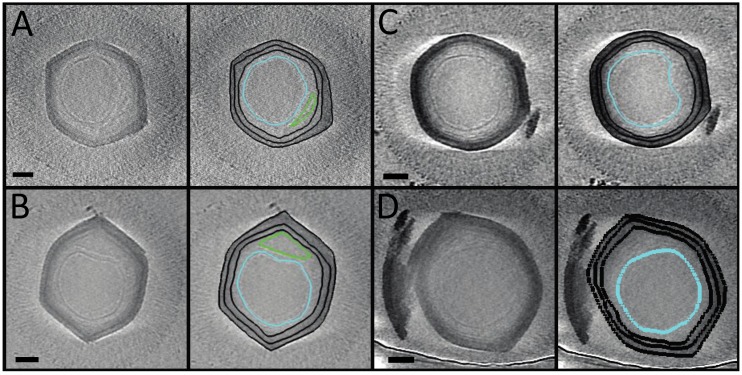
Cryo-electron tomograms of SMBV particles. These micrographs depict two-dimensional projections of three-dimensional data from four representative SMBV tomograms. Projections represent 10 slices (14.7-nm thick for (**A**,**B**) and 6.9-nm thick for (**C**,**D**) computationally combined using the slicer functionality in IMOD (3dmod). Capsid layers (black), nucleocapsid (cyan), and membrane sac (green) within the SMBV virions are highlighted in the right-hand panels. Tilt series were acquired along a tilt range of ±55**°** with tilt increments of 1–2°. Tomograms were generated using IMOD v4.7.15. Tomograms in (**A**,**B**) were collected at 4000×, and (**C**,**D**) were collected at 8000× nominal magnification. Scale bars represent 100 nm.

The structure of APMV, previously determined by cryo-EM (EMD-5039, [21]), demonstrated that APMV particles are quasi-icosahedral with one unique vertex housing the “starfish” structure used to release the nucleocapsid during genome release. Three-dimensional image reconstructions of APMV, imposing icosahedral symmetry, and/or 5-fold symmetry yielded maps clearly displayed the APMV structural features [19,21]. As SMBV is closely related to APMV [23], it was hypothesized that SMBV particles would share a similar quasi-icosahedral nature. Therefore, we attempted single-particle reconstructions of ~2800 SMBV particles using a random model computation (RMC) [37] and Auto3dem [38], as well as EMAN2 [39]. SMBV particles displayed a high degree of structural heterogeneity, as evidenced by visual inspection (Figure 1 and Figure 2), failure to obtain consistent classes using the EMAN2 classification procedure (data not shown), and results from cryo-tomography (Section 3.3, Figure 3). To eliminate the external fibers as a confounding factor for the three-dimensional reconstruction, we also attempted an RMC on fiberless SMBV particles that were present in the two-dimensional projection images. In total, we tried 100 RMCs for both the complete particle set and the subset of fiberless particles. All RMCs failed to produce a coherent icosahedral structure, suggesting that either SMBV is unlike APMV, and not quasi-icosahedral, or that we had a mixed population of icosahedral and non-icosahedral particles and were unable to distinguish between these particle types in our micrographs. If rigid, quasi-icosahedral SMBV particles are indeed present; the frequency was too low to detect them in this sample.

### 3.2. A Comparison of APMV and SMBV Particles through the Use of Cryo-Electron Microscopy

Since SMBV did not display a rigid, quasi-icosahedral capsid structure as seen in APMV, we also analyzed cryo-electron micrographs of a fiberless variant of APMV [30] (Figure 2A–C). Since a plethora of structural information is available for APMV [18,19,20,21,40], we felt that using the same experimental setup to analyze the two viruses would provide a good control to compare the shape of APMV and SMBV capsids, and to confirm that the plasticity observed in SMBV is not a result of preparation techniques. Comparing SMBV and APMV particles in the same state (both fibered or both fiberless) would be ideal, however we did not have access to identical samples. We did not have a sample of fibered APMV, and the only process, to our knowledge, which is known to defiber giant virus particles [21] is treatment with proteinase K, lysozyme, and bromelain, which does not remove the SMBV fibers. This preliminary result suggests that the composition of SMBV fibers differs from that of other members of *Mimiviridae.*

An average of the measured capsid diameters of 274 APMV particles resulted in a capsid diameter of ~499 nm, which matches the previously reported value [20] (Figure 1B). A small percentage of both APMV and SMBV particles displayed a notch-like structure at a unique vertex within the capsid (Figure 2D). This feature has been reported previously in APMV [20], although its biological function is currently unknown. APMV particles within the cryo-EM images appeared to have a much higher degree of structural homogeneity than that seen in the SMBV particles (Figure 1 and Figure 2). APMV particles within the cryo-electron micrographs were clearly quasi-icosahedral with rigid facets, consistent with the published structure [21]. SMBV particles, on the other hand, exhibited a high degree of structural plasticity (Figure 2).

### 3.3. Three-Dimensional Structural Information of the Entire SMBV Virion Was Obtained through the Use of Cryo-Electron Tomography (Cryo-ET)

With the large degree of heterogeneity displayed in the SMBV particles (see above), we were unable to generate a three-dimensional structure of the SMBV virion through the use of single particle cryo-electron microscopic analysis. Cryo-electron tomography (cryo-ET) eliminates the need to average many particles, allowing us to circumvent the heterogeneity of the SMBV particles. With a total particle diameter of ~834 nm SMBV is, to our knowledge, the largest specimen successfully imaged using cryo-ET without the use of focused ion beam (FIB)-milling [41,42], freeze fracturing [22], cryo-sectioning [43], or other techniques which are used to reduce sample thickness [44,45].

As the most abundant particle morphology, and with the fibers thought to be important for attachment, we decided to focus our cryo-ET efforts on fibered particles. We generated 20 tomograms displaying 34 fibered SMBV particles. Four representative volumes are displayed in Figure 3 (Supplemental Video 1). A representative tomogram is accessible through the Electron Microscopy Data Bank (EMDB) with the following accession number: EMD-8599. These tomograms displayed the structural features of the SMBV virion in greater detail than the single particle cryo-electron micrographs (Figure 1).

The tomograms provided enough detail to visualize several layers within the SMBV capsid, and provided further confirmation of the heterogeneity observed in our 2D projection images. In APMV, the capsid is hypothesized to consist of two layers of protein surrounding a layer of lipid, resulting in at least three visible layers within the capsid [20,46]. Like in APMV, the tomograms depicted at least three distinct layers within the SMBV capsid (Figure 2, highlighted in black in the right-hand panels), although the exact biochemical composition of these three layers is currently unknown. The average thickness of the SMBV capsid, measured at 10 locations around the capsid for 10 SMBV tomograms, was at most 43.3 ± 6.4 nm, with at least a 20.6 ± 3.6-nm separation between the outermost layers and at least 22.6 ± 3.9-nm separation between the two internal layers. Previous work has shown that the thickness of viral layers does not change according to defocus values ranging 1–8 μm [47]. In this work, we used higher underfocus objective lens settings. Therefore, we present the inter-layer spacing and the thickness of the layers as lower and upper thresholds, respectively. Capsid thickness within SMBV particles appeared to have a high degree of variation in both the thickness of the complete capsid (even within individual particles) and variation in the separation between the various capsid layers, and likely explains why we were unable to obtain a three-dimensional reconstruction from single particle analysis. As a result of this heterogeneity, we were also unable to perform meaningful sub-tomogram averaging.

Cryo-ET also provided a more detailed view of the SMBV fibers than the two-dimensional cryo-EM projection images. The external fibers appeared to be evenly dispersed throughout the SMBV virion, but they did not appear to have a uniform, rigid structure. In an attempt to determine if SMBV fibers have a helical nature, we also boxed 163 fibers from two-dimensional projections of five SMBV particles. Power spectra of these boxed fibers were generated using SPIDER as a part of the IHRSR++ workflow [48], and failed to produce a recognizable helical diffraction pattern, suggesting that the fibers are either not helical in nature, or were too heterogeneous to produce a regular pattern. Results from the tomograms show that the fibers are rather flexible and it proved difficult to extract individual fibers as 3D volumes since the fibers were very closely packed. Therefore, performing sub-tomogram averaging on extracted density from fibers was not possible with our current data set.

Like APMV, the SMBV genome is contained within an internal nucleocapsid. Sitting in the center of the virion, and containing relatively electron dense DNA, the SMBV nucleocapsid was visible within the two dimensional cryo-electron micrographs (Figure 1). However, the SMBV nucleocapsids were much easier to resolve in the cryo-electron tomograms (Figure 3, highlighted in cyan in the right-hand panels). Within the 34 SMBV particles analyzed via cryo-ET, 31 of the particles displayed clear nucleocapsid boundaries. The remainder displayed density that resembled the nucleocapsid but was not clearly discernable owing to the low contrast within the reconstructions. These nucleocapsids had an average diameter of 289.6 ± 27.8 nm, although this number is likely skewed, as some SMBV nucleocapsids were not spherical. In nine of the 31 SMBV particles with visible nucleocapsids (which corresponds to 29% of the particles with clear nucleocapsids), the nucleocapsid was deformed by ~15 nm, appearing to pull away from one capsid vertex. Where the nucleocapsid was pulled away the capsid, it resided ~75 nm away from the innermost capsid layer, as opposed to the remainder of the nucleocapsid, which was ~40 nm away from the capsid, on average. This phenomenon was also observed in APMV, with sufficient frequency to appear in the single particle reconstruction of the virus [21]. In the APMV three-dimensional reconstruction, the capsid vertex that the nucleocapsid is pulling away from houses the starfish structure. The presence of similar asymmetry in the SMBV nucleocapsid may provide further evidence that the SMBV virion also contains this so-called starfish seal at a unique vertex. The absence of nucleocapsid asymmetry in some SMBV particles was likely a result of particle orientation and is consistent with the missing wedge effect inherent in cryo-ET [45,49]. This effect limits the region of three-dimensional information available in our tomograms. In addition, three SMBV particles clearly exhibited the presence of an extra membrane sac within the virion (Figure 3A,B, highlighted in green in the right-hand panels), which was also seen in two-dimensional projections of empty capsids (Figure 1D, highlighted in cyan). The biochemical composition of this sac, and its biological function, is currently unknown. This extra membrane sac was observed in an empty APMV particle, yet was not resolved within the three-dimensional reconstruction [21], likely owing to the 5-fold averaging employed in that study.

### 3.4. A Comparison of SMBV and APMV Particles via Scanning Electron Microscopy (SEM) Revealed Differences in Capsid Regularity and Potential Viral Ultrastructure

**Figure 4:**
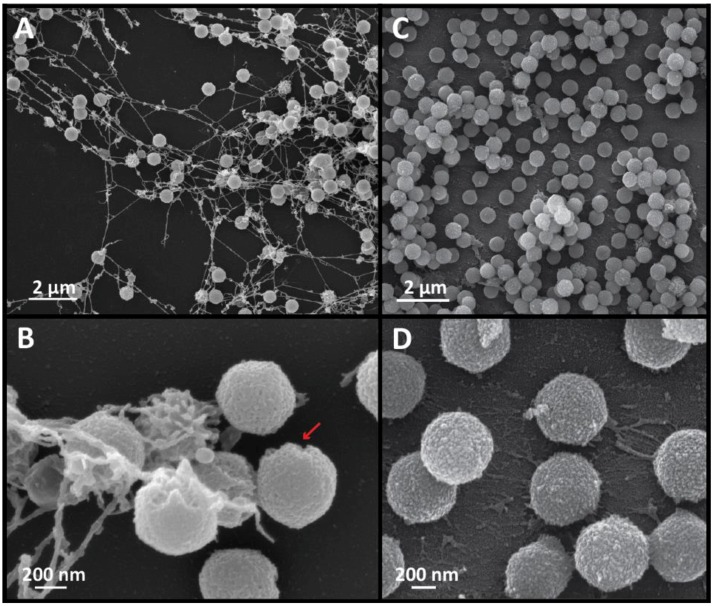
Scanning electron micrographs of SMBV (**A**,**B**) and APMV (**C**,**D**) particles. (**A**) Low magnification (7000×) field of view of SMBV particles. (**B**) Higher magnification (50,000×) image of SMBV particles. The red arrow points to a presumably fiberless region at a unique vertex of an SMBV particle, potentially revealing the location of the starfish seal. (**C**) Low magnification (10,000×) micrograph of a fiberless APMV variant [30]. (**D**) Higher magnification (50,000×) micrograph of APMV particles.

To obtain further structural information about the SMBV capsid, and to corroborate our observations from both cryo-EM and cryo-ET, we analyzed SMBV particles via scanning electron microscopy (Figure 4). To avoid dehydration of the particles, and the accompanied structural artifacts, the SMBV particles were dried using a critical point dryer prior to the sputter coating process. Low magnification SEM images revealed material stretching between the SMBV particles (Figure 4A). The composition of this material is currently unknown, but it appeared to form fibrous strings between SMBV particles. This material was consistently present, even when SEM samples of SMBV were prepared using various procedures (data not shown). It is unknown whether this material plays any role in SMBV biology.

The scanning electron micrographs also gave us some idea of the surface of the SMBV particles. Within the low magnification images, most of the SMBV particles appeared to be smooth, but a few of the particles appeared to be surrounded by a layer of “spikes” (Figure 4A,B). The “spikes” on these particles were likely external fibers that had clumped together during the critical point drying or the sputter coating processes, although this is currently impossible to determine, as we are unable to remove the SMBV fibers. Higher magnification micrographs of the SMBV particles (Figure 4B) provided greater detail of the surface of the virus and the fibrous strings. The surface of the SMBV particles did not appear to be regular when compared to that of APMV. Previous work on APMV using atomic force microscopy demonstrated a lack of fibers surrounding the starfish [40]. It appears that this may be consistent in SMBV based on surface variation at unique vertices seen in SEM data (arrow in Figure 4B highlights one such vertex).

APMV scanning electron micrographs demonstrated some connective material (Figure 4C,D) but not nearly as much as in the SMBV sample. Higher magnification micrographs of APMV viral particles provided greater detail about the surface of the APMV and SMBV particles. While the APMV particles appeared to be regular in shape and had a uniform surface, SMBV particles appeared to have variable sizes and surface uniformities.

### 3.5. Fluorescence Light Microscopy Revealed Biomolecular Composition and Ultrastructural Lattice Formation of SMBV and APMV Particles

Although techniques such as cryo-EM and cryo-ET possess near-atomic resolution in determining structures and visualizing surfaces, one can only speculate as to the exact biomolecular composition of the various virion components (fibers, capsid, etc.). Previous work has been successful in staining giant viruses using fluorescent dyes for flow cytomotery [50]. Here, we took advantage of fluorescent dyes in microscopy experiments, which allowed for the differentiation of biomolecules and provided additional details of capsid architecture that we were unable to ascertain by cryo-EM alone. To determine the positions of the various components within *Mimivirus* virions, and to perform another comparison between APMV and SMBV, we dyed the viral particles with FITC (which is amine reactive and dyes proteins) and DAPI (selective for DNA), and then visualized the dye localization through the use of fluorescence light microscopy.

**Figure 5:**
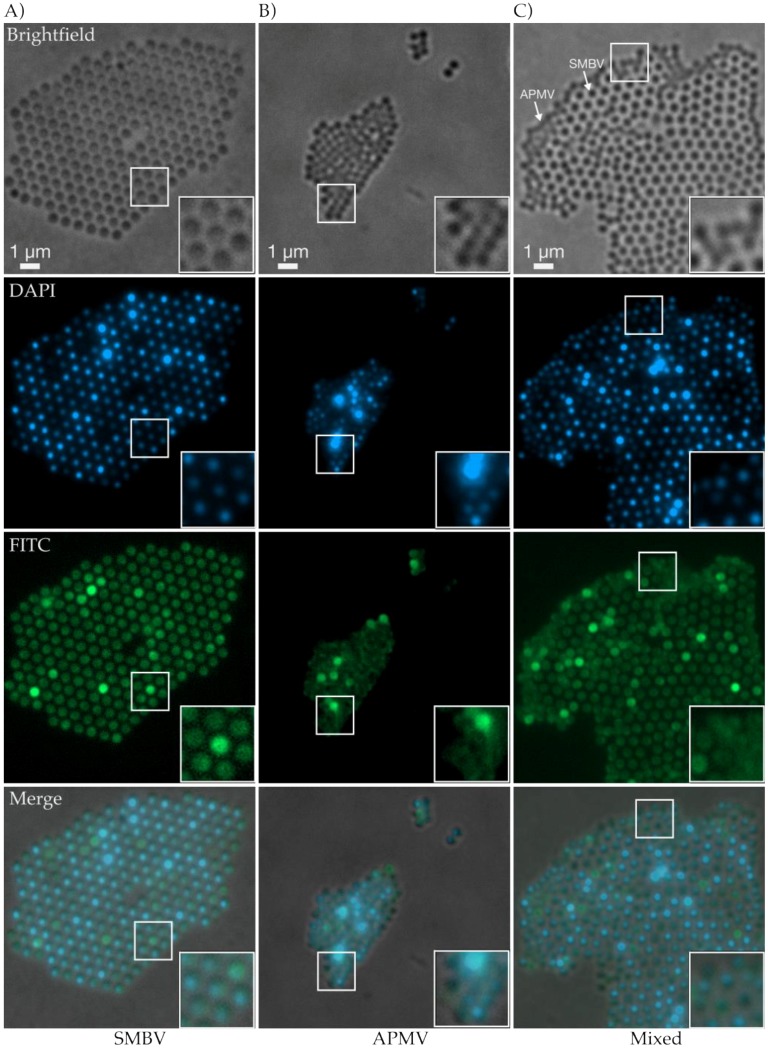
Fluorescence light microscopy of SMBV and APMV particles. (**A**) SMBV imaged via transmitted light, DAPI (4’,6-diamino-phenylindole) stain, and fluorescein isothiocyanate (FITC) stain which demonstrated defined particles and higher-order organizational characteristics (**B**) APMV imaged via transmitted light, DAPI DNA stain, and FITC protein stain, which highlighted a lack of particle definition and loose aggregation (**C**) A mixed population of SMBV and APMV imaged with transmitted light, DAPI, and FITC stains distinctly showing SMBV lattice interruption from APMV particle association.

Although we were unable to visualize the viral particles in as great of structural detail as we were able to with cryo-EM, cryo-ET, and SEM, through the use of light microscopy, we were able to view comparative similarities and differences between SMBV and APMV particles. One of the most striking results of the brightfield microscopy was the difference in organization between the two viruses. SMBV particles appeared to self-organize into large lattices, some of which were tens of microns in size (Figure 5A). This observation highlights an additional benefit to using fluorescence microscopy to visualize SMBV. In our cryo-electron experiments, we were unable to detect the presence of higher-order aggregates in the vitrified specimens as thicker areas of ice did not allow sufficient contrast in resulting micrographs, and thus were avoided during imaging. These lattices are reminiscent of the hexagonal lattices seen within bacterial cells during bacteriophage P22 infection [51]. This observation contrasts sharply with APMV (Figure 5B), which appears to form loose aggregates, lacking the rigid organization that was seen in SMBV (Figure 5A). This difference in lattice organization may be a property of the viruses themselves, but it may also be due to the lack of fibers in the APMV samples. As mentioned previously, the *Mimivirus* fibers are thought to play a role in attachment [36], so it is possible that the fibers are responsible for the organization of SMBV particles and the lack of organization within the APMV sample. The lack of organization, the abundant aggregation, and the smaller relative size of APMV particles combined to cause difficulty while imaging these particles.

As noted previously, the strength of labeling specific biomolecules, and detailing relative location within particles, is one of the main attributes of fluorescent light microscopy. DAPI DNA staining demonstrated similar attributes between particles from both viruses. For both APMV and SMBV, some particles displayed dense, brightly fluorescent DAPI staining while the other particles appeared to be more punctate (Figure 5A–C). Enlarged views of some SMBV and APMV particles (Figure 5A–C, insets) demonstrated the asymmetrically localized DAPI fluorescence within the viral particles. The DAPI fluorescence signal appeared to be smaller and contained within the brightfield and FITC signals (see below). This observation is what one would expect from a virus, with the nucleic acid genome contained within a proteinaceous capsid, and confirms that fluorescence microscopy can be used to localize virion components within giant viruses. The DAPI signal also appears to be asymmetrically localized within some SMBV capsids. This observation matches the nucleocapsid asymmetry observed in the two-dimensional projections of SMBV particles from both cryo-EM and cryo-ET.

While the DAPI fluorescence for APMV and SMBV particles appeared similar, the two viruses demonstrated stark differences when visualized for FITC fluorescence, which is amine reactive and binds proteins. The SMBV FITC fluorescence supported the brightfield observation of conjoined, self-organized particles (Figure 5A). Also, across some individual SMBV particles, the signal was particulate, demonstrating small foci of brighter fluorescence (Figure 5A, inset). Again, due to the resolution limitations of fluorescent light microscopy, it is difficult to determine the true significance of this punctate patterning of SMBV particles without further experimentation and investigation. A heterogeneously stained population is consistent with the heterogeneity observed using cryo-EM and cryo-ET as described above. APMV particles, on the other hand, lacked any detailed features under FITC fluorescence. While some APMV particles appeared to be more fluorescent than others, many of the particles lacked the clearly defined protein boundaries present in the SMBV particles, and these particles lacked the stippling feature of SMBV.

For a truly direct comparison of the APMV and SMBV samples, we combined the two viruses prior to addition of the fluorescent dyes. This mixture directly demonstrated the differences between particles within the APMV and SMBV samples, and allowed us to visualize the interaction between the two viruses. Brightfield microscopy showed a mixed lattice-aggregate of SMBV and APMV particles. The APMV particles were interspersed within the SMBV lattice (Figure 5C), and appeared to perturb SMBV particle lattices. These observations were further supported by the FITC fluorescence. The protein dye demonstrated APMV particles, which lacked a defined FITC boundary, within the larger SMBV lattices. This interspersal of APMV particles within the SMBV lattice suggests that SMBV, and potentially all *Mimiviruses*, are able to interact with other virus particles within aggregated lattices. We speculate that the giant virus-associated virophages (e.g., Sputnik, Rio Negro virus) may also be able to interact in these lattices during *Mimivirus* infections.

## 4. Discussion

In summation, the cross-platform techniques as described in this paper highlight similarities and differences between SMBV and APMV. SMBV has a larger capsid diameter (~527 nm), fiber length (~155 nm), and total particle diameter (~834 nm) than APMV (~500 nm, ~125 nm, ~750 nm, respectively), making SMBV the largest member of *Mimivirus* described to date. The major difference between APMV and SMBV appears to be the global structure of the viral capsid. APMV particles appear to be quasi-icosahedral, with rigid sides and a unique vertex that houses the starfish complex, consistent with previously published reports. SMBV, on the other hand, does not appear to share the same degree of rigidity and a quasi-icosahedral architecture with rigid facets is less obvious. Instead, SMBV exhibits a much higher degree of structural variance. For example, in the cryo-EM images, APMV particles appear to be more regular in shape and have fewer structural variations than the SMBV particles. In the SEM images, APMV particles appear to have a smoother capsid surface and fewer structural irregularities. SMBV particles form self-organized lattices within the fluorescence micrographs whereas APMV particles tend to randomly aggregate. In cryo-EM, cryo-ET, and fluorescence micrographs SMBV particles show an asymmetrically-localized nucleocapsid, which varies in structure from particle to particle. Future work to make use of advanced light microscopy techniques (such as super-resolution microscopy) will help to elucidate if these are indeed common features among giant viruses and will provide additional insight that cannot be gained from electron microscopy alone.

There are over 50 mimiviruses isolated and characterized to date. Recently, a pan-genome analysis compared SMBV, APMV, and others [24]. Key results reveal that the genome of SMBV is most similar to APMV, and retains high similarity with other *Mimiviruses* such as Oyster virus (OYTV) and Amazonian virus (AMAV). This pan-genome analysis of Brazilian *Mimivirus* group A showed that a total of 58 clusters consisting of 179 paralogous proteins were identified in SMBV, which is similar to APMV, and reciprocal best-hit analysis identified 917 orthologous proteins shared between these viruses. The four predicted capsid proteins in SMBV have 98–100% identity to those known in APMV. Previous predictions indicate that the APMV major capsid protein “L425” is likely to have a jelly-roll domain [21]. It is tempting to predict that the SMBV major capsid protein will have a similar structure. However, making structural predictions regarding the capsid protein based solely on the genetic material is difficult at best. For example, introns in the mimivirus capsid protein gene have been shown to complicate genomic predictions, and mass spectrometry and recombinant expression systems were required to fully characterize this gene product [52]. The SMBV capsid protein gene has up to three introns (Genbank AHJ40114.2). We can conclude that there are sufficient differences in the global architecture of SMBV and APMV. Therefore, it follows logically that there will likely be some differences in the structural protein building blocks that form the native virions. Further detailed biochemical and structural experiments of the SMBV capsid proteins are needed to dissect these differences at the molecular level.

## Supplementary Materials

The following are available online at www.mdpi.com/1999-4915/9/2/30/s1, Video S1: Z-slices of a representative SMBV tomogram (central section depicted in Figure 3B).

## Abbreviations

SMBV	Samba Virus
APMV	*Acanthamoeba polyphaga mimivirus*
Cryo-EM	Cryo-electron Microscopy
Cryo-ET	Cryo-electron Tomography
SEM	Scanning Electron Microscopy
TEM	Transmission Electron Microscopy
RMC	Random Model Computation
